# The Association between Idiopathic Pulmonary Fibrosis and Obstructive Sleep Apnea: A Systematic Review and Meta-Analysis

**DOI:** 10.3390/jcm11175008

**Published:** 2022-08-26

**Authors:** Filip Franciszek Karuga, Piotr Kaczmarski, Bartosz Szmyd, Piotr Białasiewicz, Marcin Sochal, Agata Gabryelska

**Affiliations:** 1 Department of Sleep Medicine and Metabolic Disorders, Medical University of Lodz, 92-215 Lodz, Poland; 2 Department of Pediatrics, Oncology, and Hematology, Medical University of Lodz, 91-738 Lodz, Poland; 3 Department of Neurosurgery, Spine and Peripheral Nerves Surgery, Medical University of Lodz, 90-549 Lodz, Poland

**Keywords:** OSA, IPF, fibrosis, prevalence, comorbidity

## Abstract

The prevalence of obstructive sleep apnea (OSA) has greatly increased in recent years. Recent data suggest that severe and moderate forms of OSA affect between 6 and 17% of adults in the general population. Many papers are reporting the significantly increased prevalence of OSA in patients suffering from fibrotic diseases, including idiopathic pulmonary fibrosis (IPF). Therefore, we performed a systematic review and meta-analysis regarding the dependency between IPF and OSA. Due to the lack of papers focusing on IPF among OSA patients, we focused on the prevalence of OSA among IPF patients. In the search strategy, a total of 684 abstracts were identified, 496 after the removal of duplicates. After the screening of titles and abstracts, 31 studies were qualified for further full-text analysis for eligibility criteria. The final analysis was performed on 614 IPF patients from 18 studies, which met inclusion criteria. There were 469 (76.38%) IPF patients with OSA and 145 (23.62%) without. The mean age varied from 60.9 ± 8.1 up to 70.3 ± 7.9. The obtained prevalence was 76.4 (95% CI: 72.9–79.7) and 75.7 (95% CI: 70.1–80.9) for fixed and random effects, respectively. The median prevalence of OSA among non-IPF patients for all the ethnics groups included in this study was 16,4% (IQR: 3.4%–26.8%). The study provides strong evidence for the increased prevalence of OSA in IPF patients when comparing with the general OSA prevalence.

## 1. Introduction

Obstructive sleep apnea (OSA) is one of the most common sleep disorders in the world, with an estimated prevalence between 6–17% of adults in the general population in its moderate or severe forms [1]. According to recent literature, OSA affects up to 1 billion people worldwide in its all forms and this number is still rising [2]. OSA is a common chronic sleep-related breathing disorder characterized by recurrent episodes of apneas and hypopneas due to airway collapse or obstruction during sleep. It leads to intermittent hypoxia, hypercapnia, arousals, and sleep fragmentation. The golden standard of OSA diagnosis is polysomnography (PSG) and the golden standard treatment is the application of continuous positive airway pressure (CPAP) [3,4]. Many papers are reporting the significantly increased prevalence of OSA in the group of patients suffering from fibrotic diseases, including systematic sclerosis, liver fibrosis, and idiopathic pulmonary fibrosis (IPF) [5,6,7]. Fibrosis is defined as the accumulation of excess extracellular matrix components. The fibrotic process is usually progressing, which in the final stage can lead to pulmonary failure and death. Pathogenesis of fibrosis is complex; there are many factors influencing the recruitment, differentiation, proliferation, and activation of the extracellular matrix [8]. IPF is a fatal, chronic, and progressive disease of mostly unknown pathogenesis, which belongs to the group of interstitial lung disease (ILD) [9]. Based on gender, age, forced vital capacity, and diffusing capacity of the lung for carbon monoxide, three different stages, I, II, and III, of IPF were proposed. Stage III is associated with the worst prognosis and almost 40% risk of death during the first year of disease. IPF is characterized by increased matrix deposition, progressive scarring of the lungs, and destruction of lung architecture. In the pathophysiology of IPF, the inflammatory and fibrotic processes play a key role. The common elements of pathophysiological processes of both diseases might include the cytokines such as interleukin 8, interleukin 6, or hypoxia-inducible factor-1 (HIF-1α) [10]. HIF-1α regulates the response to hypoxia in OSA patients by activation of a vast number of genes such as vascular endothelial growth factor (*VEGF*), erythropoietin, and aldolase-A [11,12,13,14]. In the study by Epstein Shochet et al., it was found that HIF-1α signaling pathway is also overexpressed in IPF extracellular matrix. HIF-1α activation was followed by overexpression of fibrosis-inducing genes such as *SERPINE1*, *TIMP1,* and *VEGF*. Additionally, HIF1α signaling pathway was inhibited with nintedanib (tyrosine kinase receptor antagonist that suppress collagen formation and is used to treat IPF), what led to the blockage of pro-fibrotic processes in the extracellular matrix [15]. At the present level of knowledge, it is difficult to access if the IPF is a risk factor for OSA or if this relation is bidirectional. Therefore, we performed a systematic review and meta-analysis regarding the dependency between IPF and OSA.

## 2. Materials and Methods

We conducted a systematic review in accordance with Preferred Reporting Items for Systematic reviews and Meta-Analyses (PRISMA).

### 2.1. Eligibility Criteria

Studies included in this review met the following criteria: (1) adult participants of either sex, (2) mean/median age of participants > 60 years old, (3) participants with a diagnosis of OSA based on the apnea–hypopnea index (AHI) > 5 or ≥5, (4) participants were evaluated for sleep-disordered breathing by objective method–in laboratory PSG and/or with at home PSG, and/or polygraphy (5) participants were evaluated for IPF, (6) the study was reported in English [16], (7) cross-sectional, cohort, case–control studies, and randomized controlled trials were included

The age criterium for study inclusion is results from the great prevalence of IPF in older patients with a median age at presentation around 66 years [17].

### 2.2. Information Sources

The systematic literature search was performed with the use of online databases including: “PubMed” (on PubMed.gov (accessed on 4 October 2021)), “PubMed Central” (PMC), “Embase” (on Ovid), and “Scopus” (on Scopus.com (accessed on 4 October 2021)). Paper extraction from databases was performed on October 4th, 2021. The detailed search strategy is included in Table 1.

### 2.3. Study Selection

**Figure 1:**
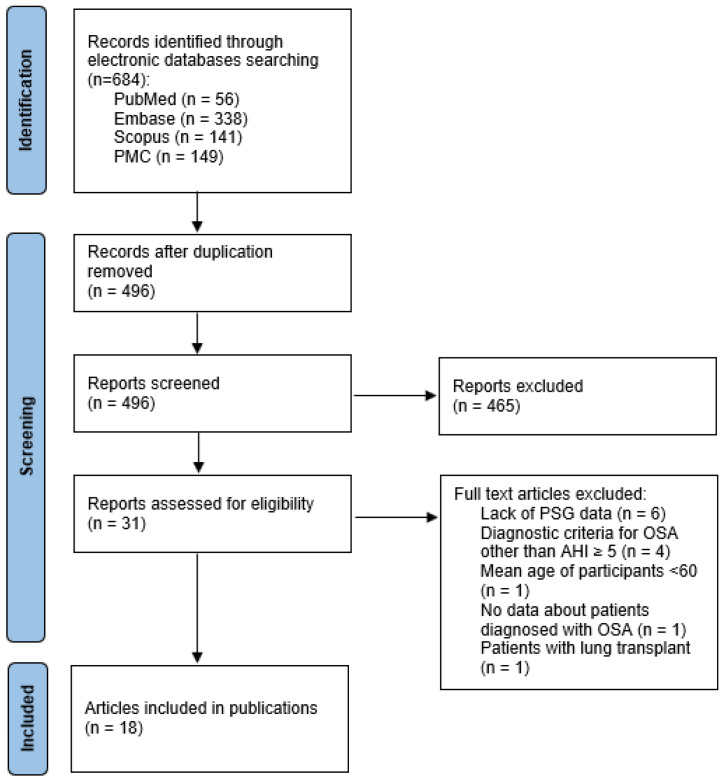
PRISMA flow chart. Legend: AHI, apnea–hypopnea index; PSG, polysomnography; PMC, PubMed Central; OSA, obstructive sleep apnea.

In the search strategy, a total of 684 abstracts were identified, 496 after the removal of duplicates. After the screening of titles and abstracts, 31 studies were qualified for further full-text analysis for eligibility criteria. Six studies were excluded due to the lack of polysomnographic data [18,19,20,21,22,23]. Four articles included different diagnostic criteria for OSA–other than AHI ≥ 5 [24,25,26,27]. The mean age of participants of one trial was <60 years old [28]. One publication included participants after the lung transplant; this situation disturbs the pathophysiological pathways linking OSA and IPF [29]. There was a lack of data about patients with OSA in one study [30]. The total number of 18 trials met our inclusion criteria and were included in the analysis (Figure 1).

### 2.4. Data Extraction

A standardized form was employed for data extraction from the aforementioned 18 studies. This stage was performed manually by P.K. with further validation by F.F.K. The following data was extracted: PSG results, age, the study characteristics (e.g., the nature of the comparison group) as well as data pertaining to source characteristics of included studies (e.g., publication status, year).

### 2.5. Statistical Analysis

The Freeman–Tukey transformation was employed to calculate the weighted summary proportion in both the fixed and random effects models. Moreover, we conducted tests assessing heterogeneity and the publication bias (Egger’s test and Begg’s test juxtaposed with funnel plot). All calculations were made with the usage of MedCalc Statistical Software version 19.1.2 (MedCalc Software, Ostend, Belgium).

## 3. Results

### 3.1. Characteristics of Eligible Studies

The final analysis was performed on 614 IPF patients from 18 studies. There were 469 (76.38%) IPF patients with OSA and 145 (23.62%) without. The mean age varied from 60.9 ± 8.1 in the study by Pihtili et al. [31] to 70.3 ± 7.9 in the paper of Mermigkis et al. [32] (see Table 1 for details).

### 3.2. Prevalence of OSA in IPF Patients

The performed test for heterogeneity revealed Cohran’s Q of 40.2, I^2^ of 57.7% (95% CI: 28.6%–74.9%) and *p*-value of 0.001. As the heterogeneity was significantly observed, the random effects model was the preferred model. The publication bias was assessed using the funnel plot accompanied by both Egger’s test and Begg’s test (see Supplementary Figure S1). The tests revealed the following results: intercept of -0.95 (95% CI: −3.67–1.77; *p*-value = 0.470) for the first one and Kendall’s Tau of -0.066 and *p*-value of 0.701 for the second one (see Supplementary Tables S1 and S2). Obtained results did not reveal significant publication bias.

**Figure 2:**
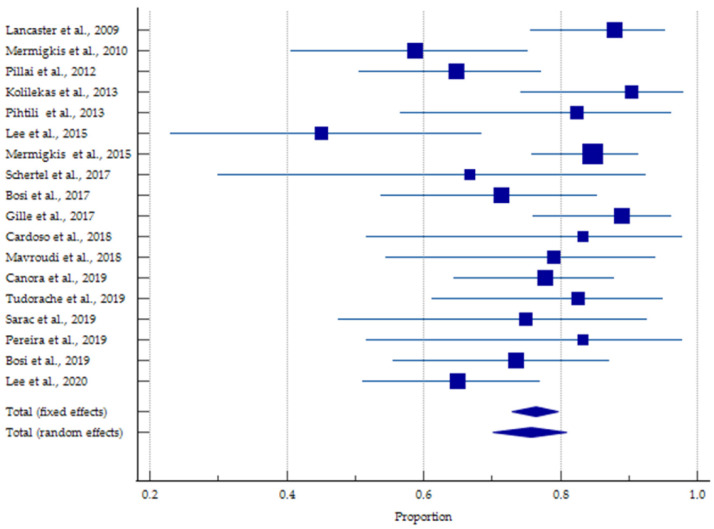
Forest plot of the prevalence of obstructive sleep apnea among patients with idiopathic pulmonary fibrosis patients. Each square represents the effect estimate of a single publication, while horizontal lines represent a 95% confidential interval. The size of squares is proportional to the weight of each paper in the meta-analysis [5,31,32,33,34,35,36,37,38,39,40,41,42,43,44,45,46,47]. Exact data are available as Supplementary Table S3.

In the current meta-analysis, we focused on the prevalence of OSA among IPF patients, due to the lack of papers focusing on IPF among OSA patients. The appropriate forest plot is presented in Figure 2. The obtained prevalence 76.43 (95% CI: 72.92–79.68) and 75.72 (95% CI: 70.15–80.90) for fixed and random effects, respectively.

### 3.3. Prevalence of OSA in Ethnically Matched Population

The prevalence of OSA significantly differs according to ethnicity, age, comorbidities, BMI, and other factors. In Table 2 we presented the ethnically matched OSA prevalence in non-IPF patients in order to have a more reliable comparison. The median prevalence of OSA among non-IPF patients for all the ethnics groups included in this study was 16.35% (IQR: 3.44%–26.77%).

## 4. Discussion

In this systematic review, we summarize the results of 18 clinical studies including 614 IPF patients. The studies included in the analysis provide evidence about sleep breathing comorbidities in interstitial lung diseases with the subgroup of IPF. Our purpose was to focus on the possible correlation between OSA and IPF. Due to the lack of studies about the prevalence of IPF in OSA patients we mainly concentrated on the prevalence of OSA in IPF patients. To increase the quality of the systemic review, inclusion criteria used in the literature screening stage (AHI ≥ 5 or AHI > 5) needed to be determined by standard PSG examination or home PSG examination.

OSA is one of the most common sleep respiratory diseases. Its prevalence estimation ranges from 6–17% in the general adult population [1]. Moreover, the median OSA prevalence in the adult ethnically matched population was 16.35%. Our results indicate that OSA is more common in the group of IPF patients than in the general population with a prevalence estimation of 75.72 (95% CI: 70.15–80.90).

IPF is a relatively rare pulmonary condition correlated with a considerable number of comorbidities such as pulmonary hypertension, pulmonary embolism, lung cancer, gastroesophageal reflux, and cardiovascular comorbidities [57,58]. Another common condition related to IPF is OSA [39]. This relation is interesting and problematic for a variety of reasons. Firstly, the interaction does not occur only at the molecular level, for example, through the HIF-1α signaling pathway. There have been some reports regarding the influence of decreased lung capacity in restrictive pulmonary diseases such as IPF leading to destabilization of upper airways and as a result to development of OSA [59,60]. Another proposed pathophysiological pathway linking IPF and OSA is the respiratory arousal threshold (ArTH). ArTH in OSA is defined as the level of airflow limitation preceding micro-arousals. Low ArTH is a common phenomenon in OSA and may be responsible for numerous arousals and high arousal index leading to sleep fragmentation preventing progression to deeper sleep stages. Shallow sleep stage S1 with high arousal index prevents the stabilization of respiratory drive and its proper synchronization with pharyngeal dilatator muscles which on-time activation is crucial in preventing upper airways collapse during inspiration [61]. Interestingly, similarly to OSA, patients with IPF have elevated arousal index which may be a risk factor for OSA due to the aforementioned pathologic pathway of desynchronization between respiratory center drive and function of pharyngeal dilatator muscles [35,62].

IPF is one subtype of ILD. Even though there are a number of studies providing evidence that OSA is correlated with ILD [31,63,64], we decided to select patients suffering only from IPF due to significant differences between ILDs in the context of OSA. IPF is a fibrotic disease in which pathology specifically affects the lungs, while many other ILDs such as sarcoidosis and connective tissue diseases may affect other systems or organs leading to disturbed sleep breathing [65,66], which is not caused by dysfunctional lung ventilation [60,67]. An example of that situation may be a primary Sjogren syndrome manifesting as ILD and affecting the OSA through increased upper airway surface lining tension [68,69,70]. The large group of studies in our review contains information regarding several types of ILDs; however, we extracted data about patients with IPF only.

Apart from the complex and not fully discovered pathogenesis of OSA in patients with IPF, these conditions are linked with each other through other common comorbidities, which makes IPF management even more challenging and complex. Pulmonary hypertension is one of the most important and severe manifestations of IPF [71]. IPF patients with comorbid OSA may present with increased risk for pulmonary hypertension (PA). The latest reports provide the evidence that nocturnal hypoxemia in OSA may cause the aggravation of pulmonary hypertension in the course of IPF which results in worse prognosis and increased mortality of IPF patients [24,35]. IPF itself leads to PA due to several pathologic changes, e.g., distortion of pulmonary ultrastructure causing ventilation–perfusion mismatch with progressing alveolar hypoventilation and reduction in cross-sectional areal of pulmonary capillaries [72]. Alveolar hypoventilation induces constriction of pulmonary capillaries and in this mechanism increases vascular resistance and in effect mean pulmonary arterial pressure. Effect of OSA due to intermittent nocturnal alveolar hypoxia may be additive, including the effect of hypoxia on concentration of vasoactive agents [72]. It has been described that in conditions of intermittent hypoxia endotelin-1 is elevated and therefore acts as a vasoconstrictor on smooth muscles cells in pulmonary arteries leading to increased pulmonary blood pressure [73,74]. Hypoxia conditions may also cause suppression of endothelial nitric oxide (NO) synthase and therefore impairment of vasodilation dependent from endothelium [75]. There are some literature reports providing evidence that alveolar exhaled NO is reduced in the group of OSA patients [75]. Correlation of several pathogenetic pathways in hypoxemic conditions may lead to pulmonary hypertension, which is crucial factor for worse IPF prognosis [76]. Gastroesophageal reflux is another common comorbidity that could be a result of the coexistence of IPF and OSA and is related with acute exacerbations in IPF [77]. Moreover, OSA is a known risk factor for cerebrovascular incidents and may be a life-threatening disease for IPF patients with an already increased risk of vascular diseases [78]. The results of our review are in line with current literature reports on this topic [39,79].

The significant prevalence of OSA in IPF patients raises a question about the impact of OSA treatment on the course of IPF. Continuous positive airway pressure (CPAP) therapy is the most effective treatment for OSA. There is an ongoing discussion about the effectiveness, safety, and acceptance of CPAP therapy in IPF patients [80]. Although CPAP is not an easy therapy to implement, it has a lot of positive results when it comes to quality of life and prevention of comorbidities typical for OSA. Intermittent sleep oxygen desaturation is an important pathogenetic factor in both diseases and a survival predictor in IPF [35]. CPAP therapy could mitigate nocturnal desaturations and therefore may improve the prognosis of IPF [81]. Effective CPAP therapy is proven to improve daily living activities and quality of life in patients with coexisting OSA and IPF [82]. Another positive effect of CPAP therapy may be a positive effect in decreasing pulmonary hypertension correlated with OSA and potentially in IPF–OSA patients, although this effect has not yet been proven [83,84]. On the other hand, CPAP therapy raises some problems with compliance in the group of OSA–IPF patients. IPF patients report chronic symptoms that can be exacerbated by the implementation of CPAP therapy, e.g., chronic nocturnal dry cough [80]. There is one study describing symptoms such as nocturnal cough, claustrophobia, and insomnia as a reason for discontinuation of CPAP therapy, although the authors added heated humidification to improve the side effects of this therapy [82]. The observations from the clinical study conclude that good CPAP treatment compliance may play an important role in the survival of OSA–IPF patients [32]. Authors of a 10-year retrospective observational study suggest that CPAP therapy has a positive effect in terms of progression-free survival and FVC (forced vital capacity) preservation only in a subpopulation of OSA–IPF patients requiring oxygen supplementation [26].

Another important problem with CPAP compliance in the group of IPF patients is oxygen supplementation. In the course of IPF, alveolar hypoxia progresses and eventually oxygen therapy is required [85]. Conceivably, if OSA is a comorbid condition, it usually leads to superimposed intermittent nocturnal alveolar hypoventilation with ensuing alveolar hypoxia with concomitant hypercapnia [86,87]. IPF patients typically have increased respiratory drive to off-set alveolar hypoxia; thus, the oxygen therapy is safe, and not endangered by increased hypercapnia [88]. Nevertheless, if they happen to have apneas during sleep, oxygen therapy can mitigate hypoxia, but cannot improve hypercapnia. Therefore, based on the pathophysiological background, it can be posited that combined oxygen–CPAP dual therapy is worth trying [26]. Unfortunately, there is still not enough prospective data regarding the safety, side effects, and indications for implementation of CPAP treatment in OSA–IPF patients [80].

Our study concentrates on the assessment of OSA prevalence among IPF patients, although there are emerging studies providing evidence about the impact of OSA on interstitial lung injury, possibly leading to the development of IPF. The results of research regarding the correlation between OSA and lung injury markers suggest that OSA patients have elevated plasma KL-6 levels (a glycoprotein localized in type II alveolar epithelial cells and a marker of lung injury) which may suggest that alveolar wall permeability is increased in OSA [89]. Additionally, authors of another study have described that KL-6 levels were significantly correlated with severity of OSA measured as AHI unlike other studied biomarkers of lung injury–surfactant protein D and C-reactive protein. The same group of researchers observed that elevated KL-6 levels in OSA patients were associated with decreased lung function tests, e.g., FVC, total lung capacity (TLC), and carbon monoxide diffusion capacity (DLCO); the changes are characteristic for lung injury and therefore restrictive lung diseases including IPF [90]. These results may indicate the role of KL-6 as a biomarker for lung injury in OSA. It has been hypothesized that lung injury in OSA may have several causes. One is traction injury caused by a repetitive decrease in lung interstitial pressure due to forced inspiration against collapsed upper airways [91]. Other proposed lung injury mechanisms in OSA include oxidative stress evoked by nocturnal hypoxemia and microaspiration in gastro–esophageal reflux, a common comorbidity in OSA and IPF [91]. In the cross-sectional analysis of 1690 adult patients, authors observed that moderate to severe OSA (AHI > 15) is associated with subclinical ILD assessed with high-attenuation areas (HAAs) and interstitial lung abnormalities (ILAs) in computed tomography. Additionally, AHI was also correlated with serum biomarkers of alveolar epithelial cells injury–surfactant protein A and matrix metalloproteinase–7 [92]. The results of these studies provide evidence that OSA may be responsible for structural changes and injury in the alveolar epithelium and lung interstitial tissue. These findings may lead to a conclusion that changes in lungs associated with OSA could predispose to the development of ILD. Further research is needed to establish the correlation between OSA and IPF.

### Strengths & Limitations

The main formal limitation of this review is the lack of registration in any prospective registry, e.g., PROSPERO, which may raise doubts about the validity of this paper, even though we performed the literature search, data extraction, and statistical analysis as described earlier in the section methods and results. We prepared our systematic review in accordance with the PRISMA protocol and good practice for statistical analysis. Another limitation of our study is the lack of longitudinal data on IPF–OSA patients. The controls in our study include inconsistent data on OSA prevalence in the general population ethnically matched with the study population.

Despite the limitation, our review is one of the first systematic approaches to establish the high prevalence of OSA in IPF patients. The results of this research may suggest that IPF–OSA patients as a separate clinical group might benefit from a more personalized diagnostic process and treatment.

## 5. Conclusions

In conclusion, this review is a summary of numerous literature reports regarding the relationship between OSA and IPF. The study provides strong evidence for the increased prevalence of OSA in IPF patients. Yet, it is important to highlight that no precise data about the nature and reasons for this relationship were available. Further research in this field can lead to a better understanding of described correlation whereby facilitation of complex diagnostic and treatment processes.

## Figures and Tables

**Table 1:** Detailed search strategy for primary literature search. IPF, idiopathic pulmonary fibrosis; OSA, obstructive sleep apnea.

Database	Search Query
PubMed (on PubMed.gov (accessed on 4 October 2021))	(“Idiopathic Pulmonary Fibrosis”[Mesh] OR “IPF”) AND (“Sleep Apnea, Obstructive”[Mesh] OR “OSA”)
PubMed Central (PMC)	“OSA AND IPF”
Scopus (on Scopus.com (accessed on 4 October 2021))	(TITLE-ABS-KEY (obstructive AND sleep AND apnea OR osa) AND TITLE-ABS-KEY (idiopathic AND pulmonary AND fibrosis OR ipf)) AND (LIMIT-TO (SUBJAREA, “MEDI”))
Embase (on Ovid)	1# idiopathic pulmonary fibrosis.mp. or exp fibrosing alveolitis 2# IPF.mp. 3# 1 OR 2 4# sleep apnea syndrome.mp. or exp sleep disordered breathing 5# (sleep adj3 (apnea or apnoea or hypopnea)).ti,ab. 6# OSA.mp. 7# 4 OR 5 OR 6 8# 3 AND 7

**Table 2:** **Characteristics of eligible studies.** BMI, body mass index; CI, confidence interval; FVC, forced vital capacity; IQR, interquartile range; IPF, idiopathic pulmonary fibrosis; ND, no data available; OSA, obstructive sleep apnea. IPF severity was assessed using FVC% predicted.

Paper	Year	Mean/Median Age of IPF Patients	Mean/Median BMI	IPF–Total	IPF Severity (FVC% Predicted)	IPF–OSA	Referral OSA Prevalence in Ethnically Matched Population	Mean/Median Age of General Population Study	Reference to OSA Prevalence
**Lancaster et al.** [5]	2009	64.9	32.3	50	ND	44.0 (88.0%)	3.4%	58.0	[48]
**Mermigkis et al.** [33]	2010	65 ± 10.6	27.3±4.0	34	72.5±18.1	20.0 (58.8%)	26.8%	54.1 ± 16.1	[49]
**Pillai et al.** [34]	2012	69.2 ± 7.0	30.4 ± 6.9	54	64.0 ± 15.9	35.0 (64.8%)	3.4%	58.0	[48]
**Kolilekas et al.** [35]	2013	67.96 ± 7.88	28,7 ± 4.3	31	77.6 ± 17.1	28.0 (90.3%)	26.8%	54.1 ± 16.1	[49]
**Pihtiliet al.** [31]	2013	60.94 ± 8.11	26.4±2.0	17	79.0 ± 23.9	14.0 (82.4%)	13.7%	40.7 ± 15.1	[50]
**Lee et al.** [36]	2015	67.9 ± 12.3	28.5 ± 4.6	20	82.2 ± 15.3	9.0 (45.0%)	3.4%	58.0	[48]
**Mermigkiset al.** [32]	2015	70.3 ± 7.9	ND	92	ND	78.0 (84.0%)	26.0%	54.1 ± 16.1	[49]
**Schertel et al.** [37]	2017	67.0 (IQR: 60.0–77.0)	24.9 (IQR: 24.0–29.8)	9	58 (IQR: 56–77)	6.0 (66.7%)	19.0%	57.0 (IQR: 49.0–68.0)	[51]
**Bosi et al.** [38]	2017	68.7 ± 9.2	22.7 ± 3.7	35	72.2 ± 19.6	25.9 (71.4%)	72.0%	40.0–85.0	[52]
**Gille et al.** [39]	2017	68.8 ± 8.7	28 ± 3.5	45	72.8 ± 20.3	40.0 (88.9%)	4.9%	46.8	[53]
**Cardoso et al.** [40]	2018	>60.0	<30.0	12	86.1 ± 18.9	10.0 (83.3%)	0.9%	62.8 (95%CI: 61.6–64.1)	[54]
**Mavroudi et al.** [41]	2018	69.8 ± 8.1	29.2 ± 5.3	19	75.6 ± 16.2	15.0 (79.0%)	26.8%	54.10 ± 16.1	[49]
**Canora et al.** [42]	2019	69 ± 7.8	28.7	54	71.5 ± 23.2	42.0 (77.8%)	72.0%	40.0–85.0	[52]
**Tudorache et al.** [43]	2019	67.6 ± 8.7	27.7 ± 4.5	23	71.3 ± 16.4	19.0 (82.6%)	6.4%	>18.0	[55]
**Sarac et al.** [44]	2019	62.0 ± 9.4	27.9 ± 4.8	16	71.0 (62.0–85.3)	12.0 (75.0%)	13.7%	40.7 ± 15.1	[50]
**Pereira et al.** [45]	2019	67.16 ± 12.18	25.6 ± 2.9	12	86.1 ± 18.9	10.0 (83.3%)	0.9%	62.8 (95% CI: 61.6–64.1)	[54]
**Bosi et al.** [46]	2019	69.7 (IQR: 64.8–74.7)	21.9	34	75.5 (IQR: 56.8–87.0)	25.0 (73.5%)	72.0%	40.0–85.0	[52]
**Lee et al.** [47]	2020	72.5 ± 7.2	24.1 ± 3.6	57	72.4 ± 14.8	37.0 (64.9%)	23.0%	49.6 ± 7.7	[56]

## Supplementary Materials

The following supporting information can be downloaded at: https://www.mdpi.com/article/10.3390/jcm11175008/s1, Figure S1: The funnel plot was generated to assess publication bias (MedCalc Statistical Software version 19.1.2; MedCalc Software, Ostend, Belgium); Table S1: Test for heterogeneity (MedCalc Statistical Software version 19.1.2; MedCalc Software, Ostend, Belgium); Table S2: Tests for publication bias. (MedCalc Statistical Software version 19.1.2; MedCalc Software, Ostend, Belgium); Table S3: Meta-analysis for proportion—exact data.
