# Supplementary material for: The Association between Idiopathic Pulmonary Fibrosis and Obstructive Sleep Apnea: A Systematic Review and Meta-Analysis
Source: J Clin Med. 2022 Aug 26;11(17):5008. doi: 10.3390/jcm11175008 (PMC9457448; doi:10.3390/jcm11175008)
Supplement: Supplementary file 1 [file jcm-11-05008-s001.zip › jcm-1792591-supplementary.pdf]

Journal of Clinical Medicine

Supplement 1

Results:

Figures

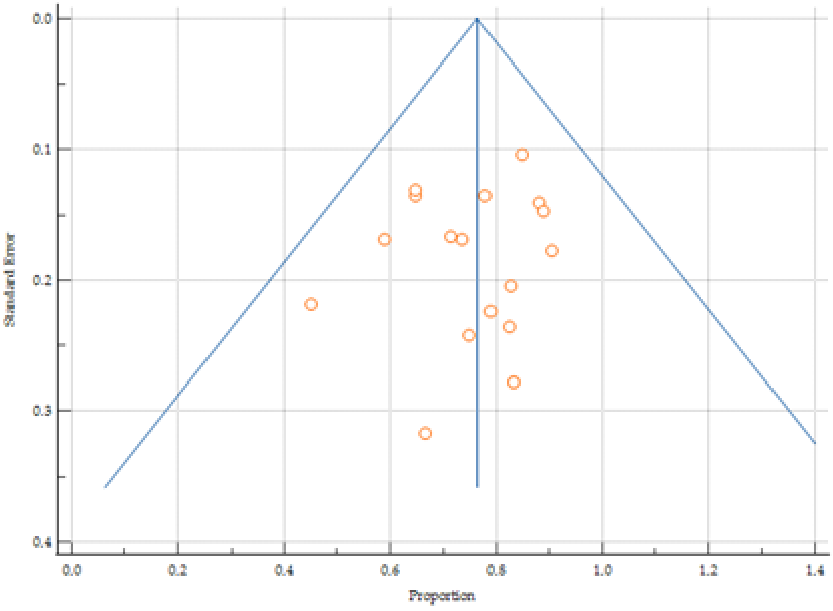

Supplementary Figure S1. The funnel plot was generated to assess publication bias (MedCalc Statistical Software version 19.1.2; MedCalc Software, Ostend, Belgium).

Tables

Supplementary Table S1. Test for heterogeneity (MedCalc Statistical Software version 19.1.2; MedCalc Software, Ostend, Belgium).

Q	40.2038
DF	17
Significance level	0.0012
I^2 (inconsistency)	57.72%
95% CI for I^2	28.62 to 74.95

Supplementary Table S2. Tests for publication bias. (MedCalc Statistical Software version 19.1.2; MedCalc Software, Ostend, Belgium).

**Egger's test**	
Intercept	−0.9506
95% CI	−3.6721 to 1.7708
Significance level	0.4697
**Begg's test**	

Kendall's Tau	−0.06623
Significance level	0.7011

Supplementary Table S3. Meta-analysis for proportion – exact data.

Study	Sample size	Proportion (%)	95% CI	Weight (%)	
				Fixed	Random
Lancaster et al., 2009	50	88.000	75.690 to 95.466	8.07	6.94
Mermigkis et al., 2010	34	58.824	40.697 to 75.353	5.54	6.02
Pillai et al., 2012	54	64.815	50.624 to 77.319	8.70	7.11
Kolilekas et al., 2013	31	90.323	74.246 to 97.958	5.06	5.80
Pihtili et al., 2013	17	82.353	56.568 to 96.201	2.85	4.32
Lee et al., 2015	20	45.000	23.058 to 68.472	3.32	4.71
Mermigkis et al., 2015	92	84.783	75.788 to 91.423	14.72	8.15
Schertel et al., 2017	9	66.667	29.930 to 92.515	1.58	2.94
Bosi et al., 2017	35	71.429	53.696 to 85.365	5.70	6.10
Gille et al., 2017	45	88.889	75.946 to 96.292	7.28	6.69
Cardoso et al., 2018	12	83.333	51.586 to 97.914	2.06	3.53
Mavroudi et al., 2018	19	78.947	54.435 to 93.948	3.16	4.59
Canora et al., 2019	54	77.778	64.400 to 87.956	8.70	7.11
Tudorache et al., 2019	23	82.609	61.219 to 95.049	3.80	5.05
Sarac et al., 2019	16	75.000	47.623 to 92.734	2.69	4.17
Pereira et al., 2019	12	83.333	51.586 to 97.914	2.06	3.53
Bosi et al., 2019	34	73.529	55.638 to 87.118	5.54	6.02
Lee et al., 2020	57	64.912	51.131 to 77.085	9.18	7.22
Total (fixed effects)	614	76.425	72.918 to 79.684	100.00	100.00
Total (random effects)	614	75.723	70.145 to 80.895	100.00	100.00
